# Tracheostomy decannulation rates in Japan: a retrospective cohort study using a claims database

**DOI:** 10.1038/s41598-022-24174-w

**Published:** 2022-11-17

**Authors:** Miho Ishizaki, Mayumi Toyama, Haruki Imura, Yoshimitsu Takahashi, Takeo Nakayama

**Affiliations:** 1 Department of Health Informatics, School of Public Health, Kyoto University, Yoshida-Konoe, Sakyo, Kyoto 606-8501 Japan; 2 Department of Infectious Diseases, Rakuwakai Otowa Hospital, Otowachinji, Yamashina, Kyoto 608-8062 Japan

**Keywords:** Health services, Medical ethics, Public health, Quality of life, Cardiovascular diseases, Respiratory tract diseases, Epidemiology

## Abstract

Despite the exponential increase in the use of tracheostomy worldwide, rates of tracheostomy decannulation are unknown. We conducted a retrospective cohort study to investigate tracheostomy decannulation rates among adult patients over a two-year period and explored factors associated with prolonged tracheostomy. A health insurance claims database including 3,758,210 people in Japan was used. The primary outcome was time to decannulation. Assessed patient and hospital factors included age, sex, emergency endotracheal intubation, disease, and hospital size. A total of 917 patients underwent tracheostomy, and 752 met the eligibility criteria. Decannulation rates were 40.8% (95% confidence interval 36.8–44.9) at 3 months, 63.9% (58.4–69.0) at 12 months, and 65.0% (59.2–70.3) at 24 months. Hazard ratios of patient and hospital factors for tracheostomy decannulation were 0.44 for age (65–74 years) (95% confidence interval 0.28–0.68), 0.81 (0.63–1.05) for female sex, and 0.59 (0.45–0.76) for emergency endotracheal intubation. Cerebrovascular disease, head injuries, and cardiac arrest had lower hazard ratios compared to other diseases. Decannulation rates among adult patients in Japan increased rapidly up to 3 months after tracheostomy, reaching a plateau after 12 months. Older age, female sex, emergency endotracheal intubation, cerebrovascular disease, head injuries, and cardiac arrest were associated with prolonged tracheostomy.

## Introduction

Tracheostomy is one of the most common surgical procedures performed in the intensive care unit (ICU)^1,2^, and its frequency has dramatically increased due to wider adoption of simple percutaneous techniques^3–5^. The number of people undergoing tracheostomy every year exceeds 100,000 in the United States^4^ and 30,000 in Japan^6^. Tracheostomy shortens the length of stay in acute care hospitals, but increases the number of patients who are transferred to long-term care facilities^3,4,7^. Resource use is extremely high for tracheostomy^7–9^, and tracheostomy reduces the quality of life (QOL) of patients.

While tracheostomy is often performed at an early stage in ICU patients^4,10,11^, previous studies reported that early tracheostomy did not improve patient outcomes, including mortality and pneumonia risk^12–14^. According to guidelines for tracheostomy, the procedure should be delayed until at least 10 days after the initiation of mechanical ventilation^12,13,15^. However, population-based database studies of tracheostomy trends in the United States reported a shortened time to tracheostomy, an increase in patients with tracheostomy transferred to skilled nursing facilities or long-term care hospitals, and a decrease in patients with tracheostomy discharged home^3,4^.

Tracheostomy management and decannulation practices require specialized knowledge and skills^10,14^. Inappropriate tracheostomy care leads to complications such as tube blockage, bleeding, cellulitis, ulceration, respiratory infection, and death^14,16^. Currently, there is no standardized decannulation protocol, and decannulation is performed according to each institution’s policy. Some hospitals have increased decannulation rates by implementing their own decannulation protocol^17–19^. However, in the absence of such efforts, some patients may be transferred to long-term care facilities without being decannulated, even if the tracheostomy is no longer needed. Moreover, little is known about the state of decannulation following discharge in acute care hospital settings. While understanding long-term decannulation rates is important in this respect, previous studies have been limited in terms of the number of facilities examined, short follow-up periods^20–22^, and narrow scope of subjects analyzed^23^. To promote appropriate tracheostomy, there is a need to better understand decannulation rates across facilities.

This study aimed to investigate tracheostomy decannulation rates among adult patients over a two-year period from the date of acute care hospitalization to the date of transfer to a rehabilitation/long-term care hospital or discharge for home treatment using a large health insurance claims database in Japan, and to explore factors associated with prolonged tracheostomy.

## Methods

### Study design and setting

We conducted a retrospective cohort study using health administrative claims data from a database established by JMDC Inc. (JMDC)^24^. JMDC has been accumulating health insurance claims received from health insurance societies since 2005. The database provides real-word data including information on diseases, drugs, medical procedures, medical materials, expenses, and hospitals. JMDC standardizes disease and drug information according to International Statistical Classification of Diseases and Related Health Problems, Tenth Revision (ICD-10) and Anatomical Therapeutic Chemical Classification System (ATC) codes. All patient data are anonymized with unique ID codes and can be tracked even if patients are transferred to a different hospital, as long as they do not change their health insurance. We used JMDC Claims Database data from January 1, 2005, to March 31, 2016. The cumulative dataset as of March 2016 included data for 3,758,210 subjects (approximately 2% of the Japanese population). Since the Japan Health Insurance Association covers healthcare for company employees and their families, the proportion of elderly people included in the JMDC Claim Database is low, with no data for those aged ≥ 75 years (age ≤ 18 years: 24%; 18–64 years: 74%; 65–74 years: 2%).

### Ethics

This study was conducted in accordance with the Ethical Guidelines for Medical and Health Research Involving Human Subjects established by the Ministry of Health, Labour and Welfare in Japan. This study was approved by the Ethics Committee of Kyoto University Graduate School and Faculty of Medicine (No. R0830). The Ethics Committee waived the requirement for informed consent because all data were anonymized and de-identified.

### Participants and outcomes

We identified adult patients (≥ 18 years) who underwent tracheostomy (procedure code: 1501062). This code applies to both surgical and percutaneous tracheostomy procedures. Patients who underwent permanent tracheostomy (procedure codes: 150107910, 150108110) were excluded.

The primary outcome was time to decannulation, defined as the time from tracheostomy to decannulation. The date of tracheostomy was the recorded date of the medical procedure “Tracheostomy” (procedure code: 1501062). The date of decannulation was defined as either the date of “Tracheostomy closure operation” (procedure code: 150108410) or 30 days after the final claim related to tracheostomy dependence (medical material code: 710010795–710010801, 710010970–710010976, 732730000–732790000, and 733830000–733890000; medical procedure code: 140009310, 140023510, and 140031430; or home tracheostomy management code: 114011110, 114050910, and 114005410). The final claim was determined based on the absence of consecutive claims related to tracheostomy dependence for more than 3 months. For patients who were transferred to a rehabilitation hospital or long-term care hospital, the date of decannulation was defined as the median length of hospitalization, since these hospitals use a lump-sum payment system and do not issue claims related to tracheostomy during hospitalization. For patients discharged from these hospitals, whether or not decannulation was performed was confirmed using claims information.

Patients who did not undergo decannulation were censored. Censored data were defined as follows: data for patients who did not undergo decannulation until the end of the study period, those who died or discontinued their claims due to unknown causes (e.g., change of health insurance), those who underwent dysphagia surgery, and those who were transferred to a rehabilitation or long-term care hospital and were not discharged until the end of the study period.

The following patient and hospital factors were considered to affect decannulation: age, sex, emergency endotracheal intubation (procedure code: 140009010), disease, and hospital size. Diseases that led to tracheostomy were identified based on ICD-10 codes. When patients had multiple diseases, the disease contracted before tracheostomy was considered to have led to tracheostomy according to medical procedure and disease codes. One of the authors, an infectious disease physician, was in charge of disease identification.

### Statistical analysis

Kaplan–Meier analysis was performed to estimate decannulation rates at 3, 12, and 24 months after tracheostomy. Stratified analyses were performed according to patient and hospital factors, and results were compared using the log-rank test. Cox proportional hazards regression analysis was performed to assess the impact of patient and hospital factors on decannulation. Results are presented as hazard ratios (HRs) with 95% confidence intervals (CIs). JMP Pro 16 was used for all analyses.

## Results

### Patient characteristics

**Figure 1:**
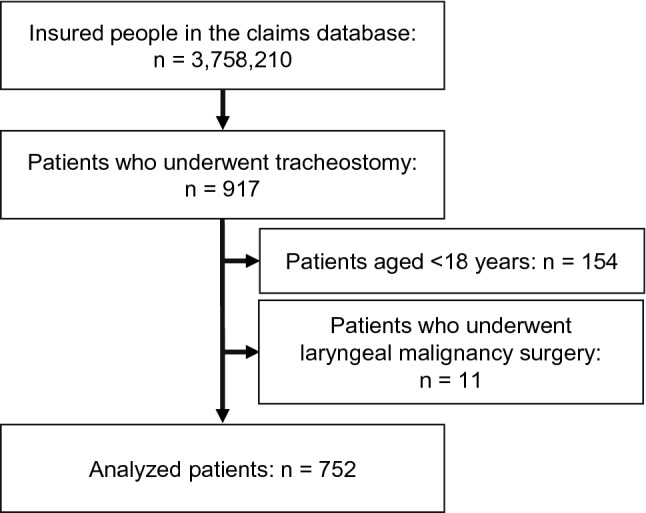
Flow chart of patient selection.

Figure 1 shows the flow chart of patient selection. Among 917 patients who underwent tracheostomy, 752 met the eligibility criteria. Patient characteristics are summarized in Table 1. Median age of participants was 53 years (IQR 44–62), and 69.7% were male. By 24 months after tracheostomy, 309 (41.1%) patients had been decannulated (40 underwent a tracheostomy closure operation and the remaining patients were confirmed to have been decannulated based on claims information), and the remaining 443 patients were censored (see Supplementary Table S1 for censored data). Prior to tracheostomy, 44.0% underwent emergency endotracheal intubation, and 235 (31.3%) patients died. Diseases leading to tracheostomy were diverse, with cerebrovascular diseases (ICD-10 codes: I60-I69; 21.9%) being the most common, followed by malignant neoplasms of lip, oral cavity, and pharynx (ICD-10 codes: C00-C14; 8.8%) and other forms of heart disease (ICD-10 codes: I30-I52; 7.3%). Other forms of heart disease included cardiac arrest (ICD-10 code: I46; 78.2%), heart failure (ICD-10 code: I50; 9.1%), cardiomyopathy (ICD-10 code: I42; 5.5%), acute and subacute endocarditis (ICD-10 code: I33; 3.6%), other cardiac arrhythmias (ICD-10 code: I49; 1.8%), and nonrheumatic mitral valve disorders (ICD-10 code: I34; 1.8%). More than 70% of patients underwent tracheostomy in large hospitals with ≥ 500 beds (Table 1).

**Table 1:** Characteristics of patients who underwent tracheostomy.

	n (%) or median (IQR)
All, n (%)	752 (100)
**Age at tracheostomy, median (IQR), years**	53 (44–62)
18–34, n (%)	92 (12.2)
35–49, n (%)	190 (25.3)
50–64, n (%)	342 (45.5)
65–74, n (%)	128 (17.0)
Sex, male, n (%)	524 (69.7)
**Diseases, n (%)**	
Cerebrovascular diseases	165 (21.9)
Malignant neoplasms of lip, oral cavity, and pharynx	66 (8.8)
Other forms of heart disease^†^	55 (7.3)
Malignant neoplasms of digestive organs	41 (5.5)
Injuries to the head	39 (5.2)
Malignant neoplasms of respiratory and intrathoracic organs	22 (2.9)
Other diseases of upper respiratory tract	22 (2.9)
Other diseases	342 (45.5)
Emergency endotracheal intubation^‡^, n (%)	331 (44.0)
**Hospital size**	
Small (20–199 beds), n (%)	40 (5.3)
Medium (200–499 beds), n (%)	185 (24.6)
Large (≥ 500 beds), n (%)	527 (70.1)

*IQR* interquartile range.

^†^Cardiac arrest, heart failure, cardiomyopathy, acute and subacute endocarditis, other cardiac arrhythmias, nonrheumatic mitral valve disorders.

^‡^Emergency unscheduled endotracheal intubation for critical care, not including testing, anesthesia, or tube replacement.

### Tracheostomy decannulation rates

**Figure 2:**
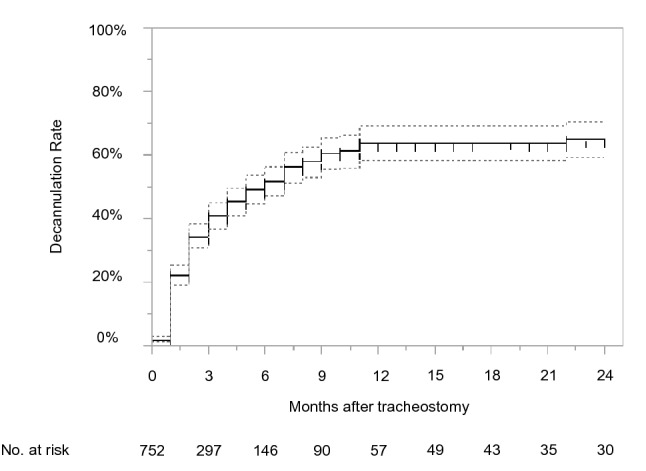
Kaplan–Meier curve for time to decannulation. Dotted lines represent 95% confidence intervals.

**Figure 3:**
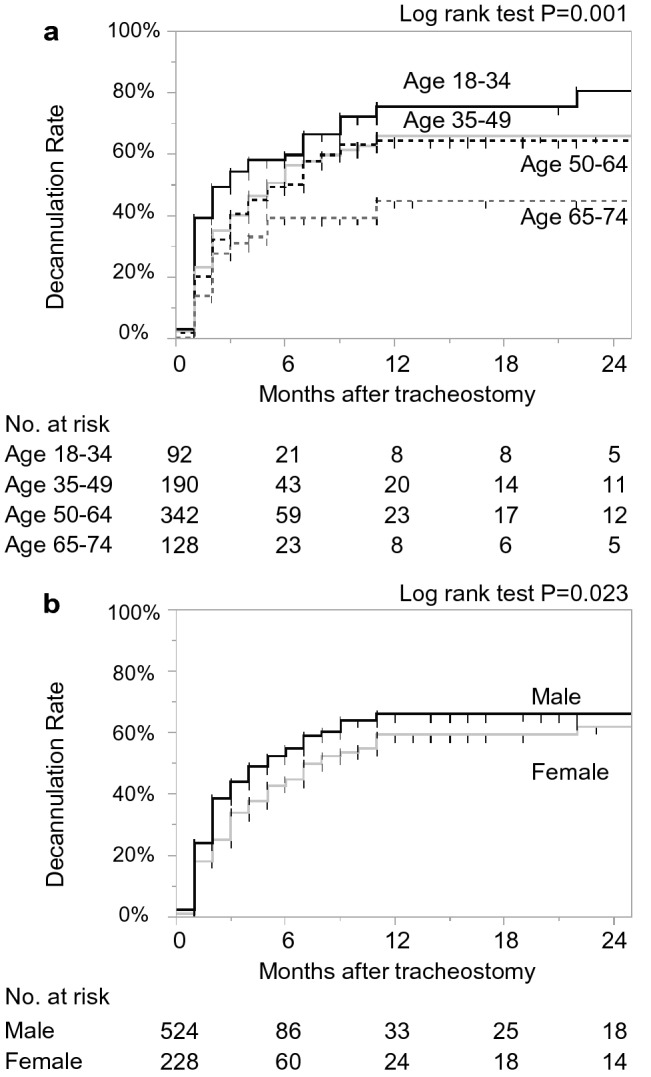
Kaplan–Meier curves for time to decannulation by (**a**) age and (**b**) sex.

**Figure 4:**
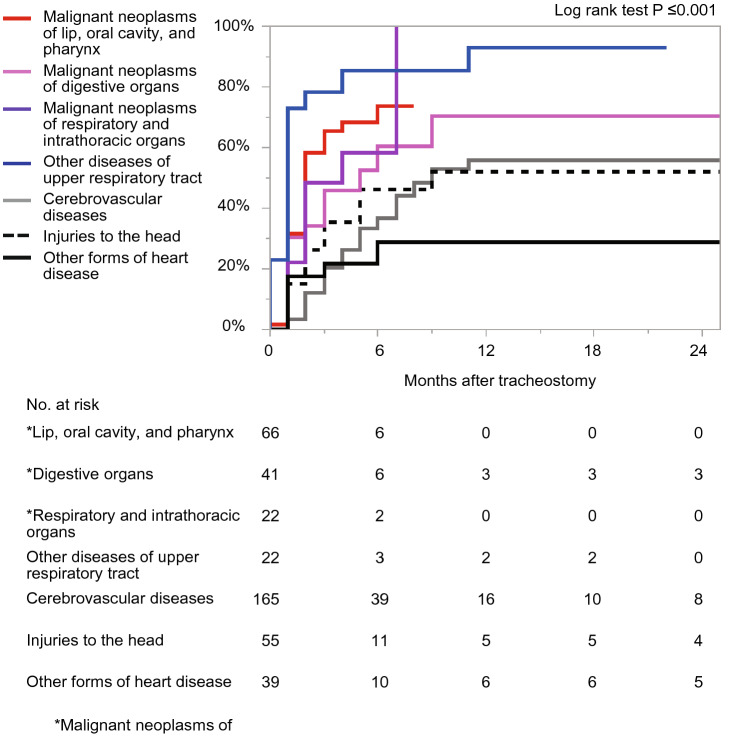
Kaplan–Meier curves for time to decannulation by diseases.

Figure 2 shows the Kaplan–Meier curve for time to decannulation. Decannulation rates were 40.8% (95% CI 36.8–44.9) at 3 months, 63.9% (58.4–69.0) at 12 months, and 65.0% (59.2–70.3) at 24 months. The results of the stratified analyses are displayed by patient and hospital factors at 12 months (Figs. 3 and 4, Supplementary Figure S1). The rate of decannulation was the lowest in the 65–74 age group (44.9%), followed by the 50–64 age group (64.5%), 35–49 age group (66.1%), and 18–34 age group (75.7%) (Fig. 3). Females had a lower decannulation rate compared to males (59.2% vs. 66.0%) (Fig. 3). Patients with cerebrovascular disease, injuries to the head, and other forms of heart disease had low decannulation rates relative to those with other diseases (55.9%, 52.2%, and 28.9%, respectively) (Fig. 4). Patients who underwent emergency endotracheal intubation had a lower decannulation rate compared to those who did not (52.0% vs. 73.6%) (Supplementary Figure S1). By hospital size, decannulation rates were 62.8% for small hospitals (20–199 beds), 46.0% for medium hospitals (200–499 beds), and 68.8% for large hospitals (≥ 500 beds) (Supplementary Figure S1).

### Factors associated with prolonged tracheostomy

Table 2 shows the results of Cox proportional hazards regression analysis for tracheostomy decannulation. Factors having an HR ≤ 1.0 included age (65–74 years: HR, 0.44 [95% CI 0.28–0.68]; 50–64 years: 0.64 [0.46–0.90]; 35–49 years: 0.75 [0.53–1.07]), female sex (0.81 [0.63–1.05]), and emergency endotracheal intubation (0.59 [0.45–0.76]). Injuries to the head (1.07 [0.57–2.02]) and other forms of heart disease (0.71 [0.33–1.53]) had lower HRs compared to other diseases.

**Table 2:** Cox proportional hazards analysis for tracheostomy decannulation.

Factors	Hazard ratio	95% CI	*P* value
**Age**			
18–34	Ref.*		
35–49	0.75	0.53, 1.07	0.111
50–64	0.64	0.46, 0.90	0.009
65–74	0.44	0.28, 0.68	< 0.001
Female	0.81	0.63, 1.05	0.110
**Disease**			
Cerebrovascular disease	Ref.*		
Malignant neoplasms of lip, oral cavity, and pharynx	2.26	1.44, 3.55	< 0.001
Other forms of heart disease	0.71	0.33, 1.53	0.385
Malignant neoplasms of digestive organs	2.09	1.20, 3.66	0.010
Injuries to the head	1.07	0.57, 2.02	0.824
Malignant neoplasms of respiratory and intrathoracic organs	1.67	0.83, 3.37	0.153
Other diseases of upper respiratory tract	3.99	2.27, 7.01	< 0.001
Others	1.72	1.22, 2.42	0.002
Emergency endotracheal intubation	0.59	0.45, 0.76	< 0.001
**Hospital size**			
20–199	1.01	0.58, 1.76	0.975
200–499	0.82	0.61, 1.10	0.179
≥ 500	Ref.*		

*Reference.

## Discussion

This study examined long-term tracheostomy decannulation rates across facilities in Japan using a large health insurance claims database, which included data after discharge from acute care hospitals. Decannulation rates were 40.8% at 3 months, 63.9% at 12 months, and 65.0% at 24 months. Factors associated with prolonged tracheostomy included older age, female sex, cerebrovascular disease, injuries to the head, and other forms of heart disease.

The decannulation rate increased rapidly up to 3 months after tracheostomy, reaching a plateau after 12 months. Decannulation is performed in acute care hospitals and rarely in long-term care hospitals or after being discharged home. A previous study conducted in an acute care hospital in Japan reported a decannulation rate of 31% at 3 months^22^; another study reported a rate of 59% in a rehabilitation hospital^20^. Our results are consistent with these reports. The average length of hospital stay in Japan is 30.6 days^25^, which is the longest among OECD countries^26^. Although the length of acute care hospital stay is fixed to up to 90 days, critically ill patients tend to remain hospitalized for a much longer period. Many of these patients are transferred to a rehabilitation hospital or long-term care hospital. In the present study, one-third of patients were transferred to a rehabilitation hospital or long-term care hospital. The problem is that, once patients are discharged from acute care hospitals, they may lose the opportunity to have their readiness for decannulation evaluated due to the lack of access to specialists and equipment. Rehabilitation/long-term care hospitals provide rehabilitation or daily care services but not specialized medical care, such as decannulation. In other words, the last opportunity for patients to have their condition and readiness for decannulation evaluated, and then undergo decannulation, is when they are still hospitalized in an acute care hospital.

The time to decannulation in Japan is relatively longer compared to other countries. Previous studies conducted in Canada, the United States, Italy, Spain, and Australia have reported shorter decannulation times and higher decannulation rates, with few adverse events^16–19,27,28^. These studies demonstrated that the time to decannulation was shortened by having specialized teams follow tracheostomy patients and developing protocols for decannulation. Patients who survive the initial acute condition but still require other intensive care are said to be chronically critically ill (CCI)^29^; the recent increase in the number of CCI patients has become a serious problem^29–32^. CCI patients are those in devastating situations, and include those with poor long-term survival, severe physical and cognitive disabilities, and significant medical costs^29–33^. A previous study on CCI found that tracheostomy, one of the clinical conditions of CCI, accounts for more than 20% of all CCI patients in Japan^32^. Intensivists need to be aware of long-term outcomes such as CCI burden^32^. Evaluating patient readiness for decannulation prior to discharge from acute care hospitals could reduce the number of CCI patients and thereby reduce the clinical and economic burden, improve patient QOL, and contribute to the establishment of a decannulation protocol.

Older age and female sex were identified as factors associated with prolonged tracheostomy. Population-based studies conducted in the United States have reported that elderly people undergo tracheostomy more often than younger people^3,4^. According to a Japanese national database, people aged ≥ 65 years account for more than 70% of all tracheostomies performed annually in Japan^6^ (see Supplementary Figure S2). Elderly people tend to have multiple diseases and are susceptible to worsening conditions, and thus, it is more likely that tracheostomy tubes are kept in place longer. Although the present study did not include patients aged ≥ 75 years, if such patients were included, we would expect a much lower decannulation rate in Japan. While the reason why more females have prolonged tracheostomy compared to males is unclear, there may exist gender disparities in treatment decisions. However, more information will be needed to verify this. Tracheostomy can reduce the QOL of patients as well as their families. When making decisions about tracheostomy, it is important that physicians understand the factors associated with prolonged tracheostomy. Further investigation of decannulation procedures is warranted to establish appropriate protocols^34^.

Cerebrovascular disease and injuries to the head were also factors associated with prolonged tracheostomy. Tracheostomy is performed in patients with a wide range of diseases, with cerebrovascular disease accounting for more than 20% in the present study. Other diseases accounted for less than 10%. Previous studies have reported that surgical patients are more likely to have early tracheostomy^4,11^, and that patients with severe brain injury may be more likely to undergo tracheostomy, with a tendency for prolonged tracheostomy. Rehabilitation is important for such patients in terms of achieving improved mobility. Readiness for decannulation should be evaluated at the acute care hospital prior to the transfer to a rehabilitation hospital.

Many patients with other forms of heart disease had cardiac arrest, and the decannulation rate among these patients was extremely low due to high mortality. This finding underscores the importance of end-of-life care in critical care settings. While advances in intensive care have saved the lives of critically ill patients, they have also created a large number of CCI patients^30^. Healthcare providers in critical care frequently encounter difficult ethical decisions about whether to save lives or deliver end-of-life care^35–37^. In the United States, treatment withdrawal is common among patients with severe brain injury due to stroke, trauma, or cardiac arrest, and those patients do not undergo tracheostomy^36^. End-of-life guidelines in critical care in Japan recommend discontinuation of life-sustaining treatment for terminally ill patients. However, it is difficult to predict patient outcomes immediately. At present, life-sustaining treatment may be provided without the need to consider these ethically difficult decisions in Japan. Treatment decisions for critically ill patients should be based on patient-centered thinking and an understanding of their values and preferences, while also avoiding overuse or underuse of services^29,35–37^. To achieve optimal end-of-life care in critical care settings, it will be important to have in place a system that makes available communication opportunities with the families of patients^35–37^. Particularly in Japan, where the super-aging of society is progressing, considering end-of-life care in critical care settings is all the more important.

There are several limitations to this study. First, the claims database did not include important factors known to affect decannulation, such as patient socio-economic status, family information, hospital characteristics, and hospital location. Second, we could not obtain certain clinical information, such as the physical condition of patients and their disease severity. Although we identified diseases from the claims data related to the procedure "tracheostomy,” this method has not been validated, and the identified diseases may differ from the actual diseases that led to tracheostomy. We did, however, identify diseases and medical procedures in consultation with researchers with experience in claims database analysis, as well as an infectious disease physician. Third, some data were censored due to patient death or transfer to rehabilitation/long-term care hospitals, from which they were not discharged until after the study period. This may have resulted in an overestimation of decannulation rates. Finally, the database is limited to company employees and their families, and people aged ≥ 75 years were not included. Therefore, our results may not be widely generalizable.

## Conclusions

Tracheostomy decannulation rates among adult patients in Japan increased rapidly up to 3 months after tracheostomy, reaching a plateau after 12 months. Older age, female sex, cerebrovascular disease, injuries to the head, and other forms of heart disease were factors associated with prolonged tracheostomy. Acute care hospitals play an important role in evaluating patient readiness for decannulation. Further investigation of decannulation procedures will be needed to establish appropriate decannulation protocols.

## Supplementary Information

Supplementary Information.

## Data Availability

The data were used under license of JMDC Inc. for the current study; therefore, the data are not publicly available. For inquiries about access to the data set used in this study, please contact JMDC (https://www.jmdc.co.jp).
