# Supplementary material for: Tracheostomy decannulation rates in Japan: a retrospective cohort study using a claims database
Source: Sci Rep. 2022 Nov 17;12:19801. doi: 10.1038/s41598-022-24174-w (PMC9672121; doi:10.1038/s41598-022-24174-w)
Supplement: Supplementary file 1 — Supplementary Information. [file 41598_2022_24174_MOESM1_ESM.pdf]

Tracheostomy Decannulation Rates in Japan: a Retrospective Cohort Study Using a Claims Database

1. Authors

Miho Ishizaki, MPH. [1], ishizaki.miho.35c@st.kyoto-u.ac.jp

Mayumi Toyama, MD, MPH, PhD. [1], toyama.mayumi.7f@kyoto-u.ac.jp

Haruki Imura, MD, MPH. [1] [2], imura.haruki.53n@kyoto-u.jp

Yoshimitsu Takahashi, DrPH. [1], takahashi.yoshimitsu.3m@kyoto-u.ac.jp

Takeo Nakayama, MD, PhD. [1], nakayama.takeo.4a@kyoto-u.ac.jp

2. Name of departments and institutions

[1] Department of Health Informatics, School of Public Health, Kyoto University, Yoshida-Konoe, Sakyo, Kyoto 606-8501, Japan

[2] Department of Infectious Diseases, Rakuwakai Otowa Hospital, Otowachinji, Yamashina, Kyoto 608-8062, Japan

Supplementary Table S1. Breakdown of censored data

	n=443
**Patient categories**	n
Patients who died	213
Patients who did not undergo decannulation until the end of the study period	85
Patients who discontinued their claims due to unknown causes (e.g., change of health insurance)	83
Patients who were transferred to a rehabilitation or long-term care hospital and were not discharged until the end of the study period	58
Patients who underwent dysphagia surgery	4

a

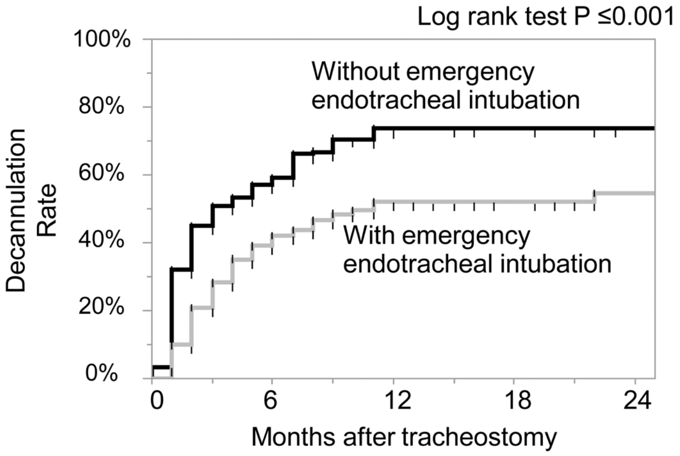

No. at risk					
Without emergency endotracheal intubation	421	67	22	17	14
With emergency endotracheal intubation	331	79	35	27	18

b

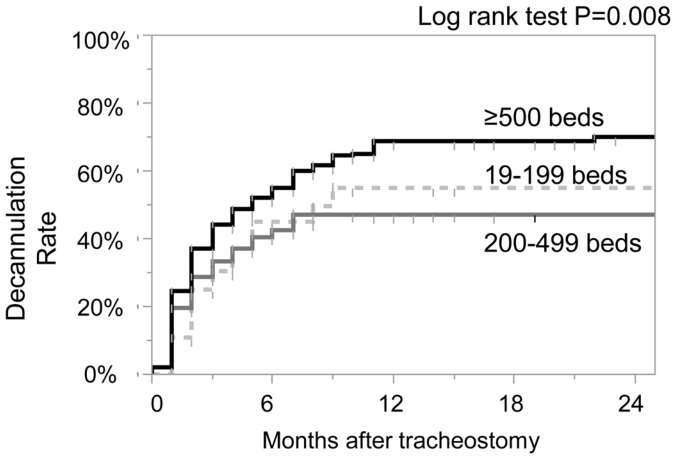

No. at risk					
Hospital beds 19-199	40	10	5	3	3
Hospital beds 200-499	185	34	15	10	8
Hospital beds ≥500	527	104	38	31	21

Supplementary Figure S1. Kaplan-Meier curves for time to decannulation by (a) emergency endotracheal intubation and (b) hospital size

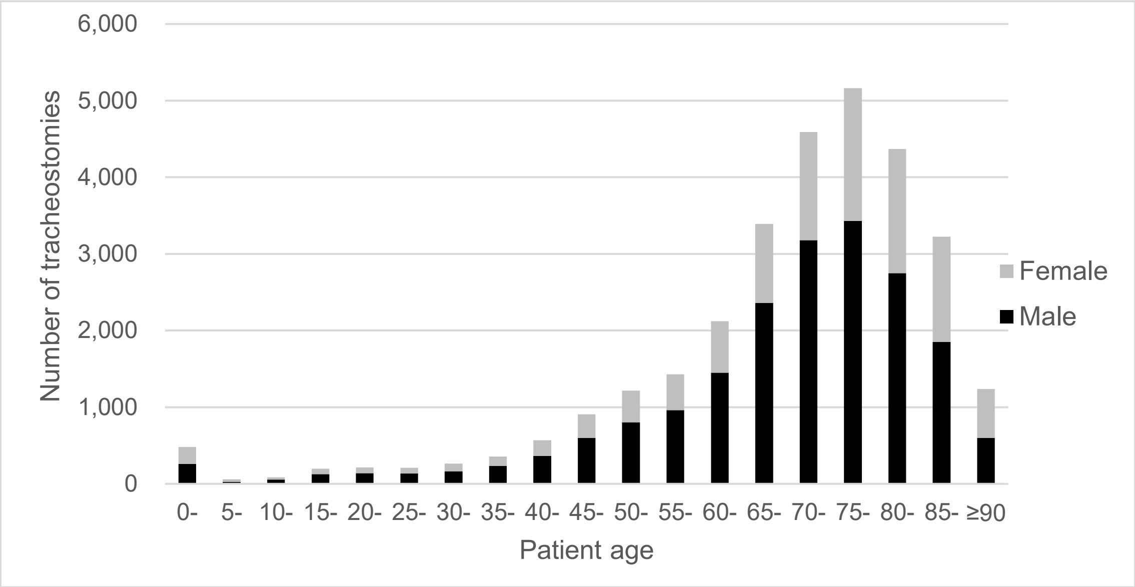

Supplementary Figure S2. Number of tracheostomies performed in Japan (April 2019 to March 2020)

This figure was created based on the 6th National Database of Health Insurance Claims and Specific Health Checkups (NDB) Open Data Japan. NDB Open Data is a national claims database provided by the Ministry of Health, Labour and Welfare, and contains claims data for almost the entire Japanese population.
